# Food Insecurity in the Rural South in the Wake of the COVID-19 Pandemic

**DOI:** 10.18103/mra.v11i12.4593

**Published:** 2023-12-26

**Authors:** Lorraine C Taylor, Seronda A Robinson, Irene A Doherty, Akeylah C Giles, Brooke E Bauer, William Pilkington

**Affiliations:** 1. North Carolina Central University, Juvenile Justice Institute; 2. North Carolina Central University, Department of Public Health Education; 3. North Carolina Central University, Julius L. Chambers Biomedical/Biotechnology Research Institute (BBRI)

## Abstract

Food insecurity in rural communities in the Southern US continues to grow, especially in the wake of the COVID-19 pandemic. Understanding the characteristics of food-insecure individuals and families in this region is critical for developing creative strategies for eliminating this health disparity issue. A food insecurity survey was given to attendees at food-security events held in several counties in one Southern US state. A descriptive analysis of food insecurity in this region is presented, and recommendations for addressing food insecurity among underserved and disadvantaged populations are suggested.

## Introduction

Food insecurity continues to impact rural communities in the United States. Aggravated by the COVID-19 pandemic, many disadvantaged families continue to struggle to obtain the amount and type of food needed to sustain their health. Federal and state efforts to provide financial assistance for needy families during the COVID-19 pandemic may have temporarily protected families and individuals from food insecurity, but recent data suggest food insecurity remains an important health concern, particularly in rural and low-income communities.^1^ Rural and low-income communities in the Southern United States are often predominately Black populations with legacies of health disparities that continue to impact the health of minority populations. Although estimates of food insecurity had steadily declined since 2011 in the US, disparities have nonetheless persisted for decades among racial/ethnic minorities.^2,4^ Pre-pandemic health disparity research indicates food insecurity as a major health disparity for Black populations.^3^ In 2021, about 20% of Blacks in the US were food insecure, compared to the 15% overall prevalence of food insecurity for the US.^2^ During the COVID-19 pandemic, research showed that food-insecure Black households were especially likely to struggle to afford food, compared to other groups. As food insecurity rates increase in these areas, the concern for public health and well-being becomes clear.

An increase in food insecurity during the COVID-19 pandemic in the US impacted the health and well-being of individuals, families, and children.^5^ In these post-pandemic times, lingering challenges persist associated with economic and food supply issues. Recent estimates suggest that food insecurity among low-income, rural regions in the US is on the rise, with families that include children being an especially vulnerable population.^6^ One cause for this upward trend in food insecurity is the lasting financial impact of the COVID-19 pandemic.^7^ As communities continue to recover from the devastating impact of the pandemic, it is important to understand changes in food insecurity that may place some individuals at greater risk for poor health outcomes.

Food insecurity impacts individuals as well as families and communities. School-aged children may be protected from family food insecurity through free or reduced-price meals in school. In 2019, approximately 14.2 billion US dollars were spent on the provision of meals to children in schools.^8^ Recent estimates show that 85% of children receiving free or reduced-price breakfast and lunch in schools live in food-insecure families,^9^ highlighting the protective function of school nutrition programs. During the COVID-19 pandemic, the closure of school cafeterias unfortunately left 30 million children without access to affordable lunches and 15 million children without access to low-cost breakfasts.^10^ For families with children, supplemental sources of food from school or other community sources ameliorate food insecurity.

Created to support scientific health disparity research and to reduce health concerns in minority populations through community collaboration and engagement, the National Institutes of Health Research Centers for Minority Populations (RCMI) awards are instrumental in ensuring researchers from Minority Serving Institutions (MSI) and Historically Black Colleges and Universities (HBCU) are on the forefront of health disparity elimination.^11^ At the RCMI Center for Health Disparities Research at North Carolina Central University (NCCU), food insecurity as a health disparity among rural and underserved communities is a major focus. The Community Engagement Core (CEC) of this RCMI focuses on connecting with rural, underserved, minority populations for research to identify effective intervention and prevention strategies that reduce health problems and promote well-being. Food insecurity in this region is a growing concern, so the RCMI-CEC embarked on a project focused on two key goals: to provide healthy foods free of cost at a series of community food distribution events and to conduct a brief assessment of food insecurity among attendees. In this article, descriptive findings from this project are presented along with recommendations for addressing health disparities within this population.

## Methods

The present project involved a collaboration between the RCMI Community Engagement Core and two key partners, the NCCU Advanced Center for COVID-19 Related Disparities (ACCORD) and the Health Equity, Environment and Population Health (HOPE) Program. These programs have collectively provided various forms of support to underserved communities. The ACCORD project was a multi-county collaborative that included the university research community, public health departments, and various community and faith-based organizations all working together to address the COVID-19 pandemic and its disproportionately negative impact on rural communities of color. HOPE is a health disparities prevention program focused on promoting health equity in historically marginalized and underserved communities in several Tier 1 counties. The Tier 1 designation indicates counties with the highest level of economic distress as determined by the State Department of Commerce.^12^ These counties are largely rural and have predominately Black populations.

A goal of the present study was to examine food insecurity in the lives of the rural, largely Black population in this region. Therefore, a descriptive study design was used, involving the collection of survey data from individuals attending a series of community food-security events in several counties around the state. Descriptive studies are especially useful for understanding the population of interest in health disparities research. To this end, a brief survey was designed for distribution to individuals attending food security events that were planned in several counties in a southern state in 2023. At these events, a variety of nutritious foods, including farm-fresh produce, were given to attendees at no cost. These events also featured information about various health disparity topics and issues, including healthy eating, long COVID-19, and demonstrations of preparing healthy snacks. Attendees were invited to participate in a short survey measuring food security.

A concerted effort was made to approach all of the adults in attendance at the food events, inviting them to complete the survey. No specific exclusion criteria were used, but attendees were informed the survey was in English. Those who agreed to participate were provided a QR code linking to the food insecurity survey in Qualtrics. Paper surveys were made available, if preferred. The completed paper surveys were later entered into the database and then shredded. Informed consent was obtained for all participants at the start of the survey. Because of the free-flowing setting of the food security events, and to minimize participant burden, it was important for the food insecurity survey to be easy to understand and complete. Given limited time for survey completion in the midst of food distribution, a shortened survey, based on the Blumberg et al. (1999) model,^13^ was utilized to capture a nuanced picture of food insecurity.

The adapted survey was comprised of 14 questions, including demographic variables and assessments of food hardship experiences. Recent food insufficiency was assessed with the question: *Has there been a day in the past 7 days when your household has not had enough food to feed everyone?* More chronic food insecurity was defined as whether children under age 18 years in the household received free or reduced-price meals at school. This was an approximate method for assessing longer durations of food insecurity, as eligibility for free or reduced-price meals in schools is a longer-term food insecurity situation. As a thank-you for completing the survey, participants were given a selection of NCCU-branded times (t-shirts, drawstring bags, etc.).

Because of the descriptive study design, statistical analyses for this project were descriptive in nature, allowing for this sample to be accurately defined and for acute and chronic food insecurity to be assessed.

## Results

Data were collected at food security events held between January and August 2023 across 6 different counties of a southern state. A total of 180 people completed the survey. Respondents were predominately African American or Black (91%) and most were women (79%) (Table 1). Participants’ ages ranged from 18–89 years with a mean age of 50.4 years and the largest proportion being older than 60 years of age (35%) (Table 1). Although 12.8% of respondents declined to report their annual income, many had very low incomes, including 19.4% earning less than US $20,000 and 20.6% earning between $20,000 to $39,999. Many respondents either lived with one other person (31.7%) or lived alone (22.2%) followed by families ranging from 3 to 8 people. Of these households, 82 (45.6%) had children younger than age 18, and among households with children, 21 (25.6%) were headed by one adult, usually a woman (n=16, 76.1%).

Descriptive findings from the food insecurity survey show that in the full sample, 48 respondents (26%) reported that there was a time when they did not have enough food to feed everyone in their household during the previous 7 days (Table 2), indicating food insufficiency. In response to the question, “What were the reasons your household did not have enough food?” half (n=24) reported it was because they did not have enough money (data not shown). Finding suggested that increasing household size generally correlated with an increase in the likelihood of experiencing food insecurity. Most of those reporting not having enough food reported annual incomes below US $40,000 (66%). There were no significant differences with respect to age.

Among the 82 households with children, 75 of them reported that the children attended school and 64% of those children received free meals at school, indicating chronic food insecurity. Families with children were more likely to receive free lunch regardless of demographics. Households with respondents 60 years of age or older were most likely to receive free lunch for the children in the household (90.9%).

## Discussion

The present study investigated the issue of food insecurity in a state in the rural southern US. Findings showed that a sizable proportion of respondents, more than 25%, experienced food insufficiency, meaning they were food insecure in the last 7 days. The shortage of money to purchase food was the major explanatory factor. This finding is consistent with evidence indicating the ongoing financial difficulties many families in this region continue to encounter following the COVID-19 pandemic,^14^ where poverty rates already tend to be above the national average.^15^ Poverty and food insecurity are inextricably linked and drive in part the prevalence of health disparities found in this region. For example, the southeast US has the highest prevalence of Type 2 diabetes in the country,^16^ a condition exacerbated by poor diet quality.^17^ Food insecurity is also a contributing factor to obesity, cardiovascular disease, hypertension, dyslipidemia, and other disparities in health outcomes, underscoring the need to ensure consistent access to nutritious foods as a critical strategy for reducing health disparities.^18,19^

Consistent with other findings, our results indicate that households with dependent children showed heightened vulnerability to being food insecure.^20^ The issue disproportionately affected families with very low incomes, specifically those earning less than US $40,000 annually. Consistent with other research, the lack of financial resources was a prominent driver of food insecurity.^21^ Studies have shown higher levels of food insecurity among rural and low-income households, particularly those with children,^6, 22^ aligning with our findings. Food insecurity estimates in southern states are consistent with the nationwide patterns seen throughout the COVID-19 epidemic, wherein children and families have met increased difficulties getting an adequate supply of basic foods due to financial constraints.^14^

This study operationally defined chronic food insecurity as children in the household receiving free or reduced-price lunch in schools. Receipt of these meals in schools could serve as an important protective factor for children, limiting the impact of food insecurity on younger members of food insecure households. During the COVID-19 pandemic, flexibility was given to school districts to ensure that children-maintained access to meals despite school closings. Following the pandemic, schools continue to struggle with high food costs and staffing shortages in their food service operations.^23^ Despite these challenges, recent efforts to expand eligibility for school meals and to offset costs to districts with increased federal funding are encouraging.^23^ Our findings suggest the importance of school meals for food-insecure families, underscoring the need for continued support for these programs.

Our findings did not reveal a higher prevalence of food insecurity among single-parent households, in contrast to other studies that show single parents have elevated risk for poverty and encountering obstacles in accessing food.^24–25^ This is likely the result of the relatively small number of single-parent households in our study. A larger, more representative sample of single-parent households would likely provide data consistent with other research findings.

Although not directly assessed in the present study, the lack of adequate access to supermarkets and grocery stores in rural regions presents a significant hurdle for individuals facing food insecurity, an important consideration for the present study. Rural households are increasingly shopping for food at convenience stores, according to recent reports.^26^ Single-parent households living in food deserts often rely on convenience stores, which typically have a limited variety of healthy food options.^27^ These convenience stores often have a limited selection of fresh fruits and vegetables, with options often restricted to just bananas and apples.^28^ Access to affordable, nutritious foods is required in order for food insecurity to be eradicated, a reality that poses challenges in many rural areas.

Several limitations in the present study should be noted. Because of the purposive, non-random sampling used for this project, the results cannot be fully generalized to all rural populations in the southern US. Data were obtained in several counties in one state, so naturally there may be differences pertaining to the histories, political landscape, geographies, and resources found in other states. With only 180 respondents, the relatively small sample size of this study limits the generalizability of our findings. Additionally, the use of the abbreviated food insecurity survey limited the ability to understand the depth and complexity of food insecurity among respondents. At the food security events where our data were obtained, detailed questions about the degree of food insecurity were not asked, as is often the case in larger studies. Nor were detailed questions asked regarding diet, availability of shopping options, or whether the respondents lived in a food desert. The food security event settings were also impacted by a variety of unique situations and logistical challenges, which may have impacted participants’ willingness or ability to complete the survey. In addition, the limited scope of this project prevented a deep analysis of links between food insecurity and specific health issues. Nevertheless, the findings from this study provide an invaluable perspective on food insecurity in the rural southern US and point to several next steps for future research and policy considerations. Future studies should employ mixed methods approaches that would include more detailed food insecurity surveys with qualitative data from focus groups or interviews with food insecure individuals. Such methods would provide valuable insights into how food insecurity is experienced in rural communities. In addition, longitudinal studies that track food insecurity patterns over time would also be helpful. Food insecurity in this particular region of the US warrants additional research.

Because the post-COVID economic challenges continue to impact families, food insecurity is likely to continue impacting families and individuals. Research examining the long-term impact of inflation periods and alterations in social safety nets on food security should continue.^29^ Our findings underscore the value of proactive measures such as the provision of free and reduced-price meals at schools. Strategies to enhance other food-related social supports such as the federal Supplemental Nutrition Assistance Program (SNAP), food pharmacies, mobile markets, and other ideas to meet the needs of rural food insecure populations more effectively must be implemented.^29^ Collaboration among public health authorities, schools, and emergency food providers is also critical. Using a comprehensive approach to address rural food insecurity and engaging with communities in culturally responsive ways is an essential strategy. Collaborative efforts are needed across health, education, and emergency food service sectors to effectively address and reduce food-related hardships in rural communities.^30^

The confluence of chronic poverty, health problems, and food insecurity in the rural southern US will continue to disproportionately affect Black communities in this post-COVID era. HBCUs have effectively led health disparity research and community engagement efforts in these communities making them a unique resource.^11^ HBCUs with medical schools are home to clinical services and translational research related to health disparity reduction in underserved, low-income communities of color. Of the four such institutions, three (Howard University, Meharry Medical College, and Morehouse School of Medicine) are located in the southern US,^31^ in areas proximal to low-income, food-insecure communities. According to Feeding America (2020), approximately 1 in 8 people face hunger in Tennessee, Georgia, and Washington DC.^32^ The expertise and strong community ties of these institutions can be leveraged to address health disparities in communities of color. HBCUs should consider partnering with community-based organizations, faith-based institutions, local health departments, and policymakers to develop comprehensive, culturally responsive interventions to alleviate food insecurity.^10^

Food insecurity elimination and the promotion of access to healthy, affordable foods should be connected to health disparity elimination research and practice. Innovative approaches are needed to accomplish this task; food distribution events such as those in the present study are one such effort to this end. Creative approaches that leverage the unique strengths of many rural environments, such as strong agricultural ties and adequate space to develop community gardens, should be considered. In addition to community garden development, other strategies like opening food pantries, farmers markets, and expanding meal delivery programs can all be used to alleviate food insecurity for rural communities.

## Conclusion

Food insecurity remains a major health disparity topic, impacting the lives of individuals and families in the Southern region of the US, many of whom are Black. Relatively high rates of poverty, economic decline, and adversities that are associated with poor health outcomes impact these rural communities, making food insecurity a complex issue that warrants additional research and creative solutions. The present study contributes to the understanding of food insecurity by providing descriptive information on food insecurity across several counties in one Southern state. Results of the present study suggest that acute and chronic food insecurity are realities for many, underscoring the need for creative approaches to reduce and eliminate this issue. Future research should better understand the nuanced experience of rural southern communities facing food insecurity by using more comprehensive measures and mixed method approaches. Finally, Historically Black Colleges and Universities are uniquely positioned to help address the complex issue of food insecurity and other health disparity topics, making them an excellent focal point for research and practice in the promotion of health and well-being in rural communities of color.

## Figures and Tables

**Table 1:** Demographics

	n	(%)
**Total**	180	(100)
**Race** ^a^		
**Black/African American**	160	(91.4)
**Other race/ethnicities**	15	(8.6)
**Gender**		
**Male**	33	(18.3)
**Female**	143	(79.4)
**Other**	4	(1.5)
**Age**		
**18–29**	17	(9.6)
**30–39**	25	(14.1)
**40–49**	40	(22.6)
**50–59**	33	(18.6)
**≥60**	62	(35.0)
**Annual Income**		
**<$20k**	35	(19.4)
**$20–39999k**	37	(20.6)
**$40–59999k**	25	(13.9)
**$60–79999k**	24	(13.3)
**>$80k**	36	(20.0)
**prefer not to answer**	23	(12.8)
**Family/household size**		
**1 – live alone**	40	(22.2)
**2**	57	(31.7)
**3**	33	(18.3)
**4**	27	(15.)
**5**	11	(6.1)
**6 to 8**	12	(6.7)
**Households with children**	82	(45.6)
**Multiple adult households**	61	(74.4)
**Single-parent household**	21	(25.6)
**Female-headed**	16	(76.1)
**Male-headed**	5	(23.9)

a Race not reported n=5

**Table 2:** Distribution of characteristics by measures of food insecurity

	Enough food for everyone in household in past 7 days				Receive free lunch			
	Not enough food		Enough food		YES		NO	
	n	(%)	n	(%)	n	(%)	n	(%)
	47	(26.1)	133	(73.9)	48	(64)	27	(36)
**Race**								
Black/African American	43	(26.9)	117	(73.1)	40	(61.5)	25	(38.5)
Other/unspecified	4	(20.0)	16	(80.0)	8	(80.0)	2	(20.0)
								
**Gender**								
Male	10	(30.3)	23	(69.7)	8	(57.1)	6	(42.9)
Female	34	(23.8)	109	(76.2)	39	(65.0)	21	(35.0)
Other	3	(75.0)	1	(25.0)	1	(100.0)	0	
								
**Age**								
18–29	5	(29.4)	12	(70.6)	5	(62.5)	3	(37.5)
30–39	5	(20.0)	20	(80.0)	14	(77.8)	4	(21.2)
40–49	14	(35.0)	26	(65.0)	15	(53.6)	13	(46.6)
50–59	10	(30.3)	23	(69.7)	4	(40.0)	6	(60.0)
≥60	12	(19.4)	50	(80.6)	10	(90.9)	1	(9.1)
								
**Household composition**								
Live alone	6	(15.0)	34	(85.0)				
Live with 1 + adults	10	(22.2)	35	(77.8)				
Children (age ≤18)	27	(32.9)	55	(67.1)				
					
**Number of people including children**								
2	13	(22.8)	44	(77.2)	7	(77.8)	2	(22.2)
3	7	(21.2)	26	(78.8)	11	(45.8)	13	(51.2)
4	11	(40.7)	16	(59.3)	11	(55.0)	9	(45.0)
5	4	(36.4)	7	(63.6)	8	(80.0)	2	(20.0)
6 to 8	6	(50.0)	6	(50.0)	11	(91.7)	1	(8.3)
